# Probiotic potential of riboflavin-overproducing Bacillus subtilis ACU-I163MR and ACU-I11MR, isolated from fermented African locust beans

**DOI:** 10.1099/acmi.0.000883.v3

**Published:** 2025-01-28

**Authors:** Theresa Awotundun, Afolake Olanbiwoninu

**Affiliations:** 1 Department of Microbiology and Biotechnology, Ajayi Crowther University, P. O. Box 1066, Oyo State, Nigeria

**Keywords:** *Bacillus subtilis*, fermented African locust beans, functional foods, probiotics, riboflavin-overproducing

## Abstract

Riboflavin (vitamin B_2_) is a water-soluble compound that plays an important role in multiple cellular functions. This study evaluates the probiotic potential of riboflavin-overproducing *Bacillus subtilis* strains isolated from fermented African locust beans. After strain improvement, *B. subtilis* ACU-I11MR and ACU-I163MR were selected due to their higher riboflavin production (0.01905±0.0005 mg l^−1^ to 0.0259±0.0077 mg l^−1^ and 0.0195±0.0054 mg l^−1^ to 0.0267±0.0013 mg l^−1^, respectively). Their safety was confirmed through haemolytic assay, antibiotic susceptibility tests and the absence of gelatinase and biogenic amine activity. Probiotic potential was assessed via *in vitro* assays including resistance to low pH, bile salts, phenol, temperature and NaCl; auto-aggregation; cell hydrophobicity; biofilm formation; antibacterial activity; and enzyme and exopolysaccharide production. Both strains were non-haemolytic and negative for gelatinase and biogenic amine activity. They showed significant viability at pH 2 (survival 85.05; 87.09%), 1% bile salts (survival 88.82; 87.64%) and 0.5% phenol (survival 48.80; 59.52%), respectively. ACU-I11MR was susceptible to 9 out of 12 antibiotics, while ACU-I163MR was 100% susceptible. The strains demonstrated strong cell surface adhesion and auto-aggregation and inhibited several pathogenic bacteria. They produce amylase, protease and exopolysaccharide and thrive under various temperature and NaCl conditions. *B. subtilis* ACU-I163MR, showing superior probiotic potential, could be a promising candidate for developing riboflavin-enriched *Bacillus*-fermented functional foods.

## Data Summary

The *gyrA* sequence data of *Bacillus subtilis* ACU-I163MR reported in this manuscript is available in the NCBI data base under GenBank accession number: PP886124. All other data are included within this article or in the associated supplementary files.

## Introduction

Riboflavin (vitamin B_2_) is a water-soluble vitamin essential for life. It plays a major role in many cellular processes, such as energy production, synthesis of haem proteins, electron transfer, oxygen transport and storage among others [1]. Unlike many plants and micro-organisms, humans cannot synthesize riboflavin and must obtain it from external sources through their diet and the production by the microbiota in the large intestine [2]. Notable food sources where riboflavin can be obtained include green leafy vegetables, milk and dairy products, yeast, cereals, meats and fatty fish [3].

Riboflavin serves as the precursor for the flavoprotein coenzymes FMN and FAD, which are the primary active forms of vitamin B2 [4]. These flavoproteins have multiple roles in the body, including coenzymes in mitochondrial metabolism, transporters of hydrogen in biological redox processes, absorption and use of iron, thyroid hormone control and the metabolism of important fatty acids in brain lipids [5]. An insufficient amount of riboflavin can cause disruption to any of these processes, which can have negative effects on the brain and other body functions.

Riboflavin is one of the six essential markers for evaluating human growth, development and nutritional health, according to the World Health Organization (WHO). According to reports, riboflavin insufficiency is common in cultures whose diets are devoid of meats and dairy products [1]. The recommended daily allowance/intake of riboflavin is 1.7 mg day^−1^, according to the European Food Information Council [6]. A balanced diet is the only way to meet this requirement. Subclinical riboflavin deficiencies are becoming more commonplace around the world due to a combination of factors such as drugs that limit absorption, certain medical conditions and increased rates of malnutrition. As a result, many nations are now recommending the mandatory fortification of staple foods as a general health strategy [1]. Microbial biosynthesis is currently the main method used in the industrial manufacture of riboflavin. The bacterium *Bacillus subtilis* and two yeast-like fungus, *Eremothecium ashbyii* and *Ashbya gossypii*, are among the producer strains [7].

*B. subtilis* have the ability to synthesize riboflavin *in situ* during the production of fermented foods [7,9]. Thus, using * B. subtilis*, which produces riboflavin, to create unique bio-enriched foods that also offer additional health advantages is a more natural and palatable option for consumers than using synthetic vitamins obtained through chemical processes [7,10].

Roseoflavin, a toxic analogue of riboflavin, can be used to cause mutations in the riboswitch regulatory region of the riboflavin (*rib*) operon. Because of these mutations, the riboflavin operon is constitutively expressed, which causes an overproduction of vitamin B_2_ [1]. As a result, bacterial strains that overproduce vitamin B2 are commonly obtained by roseoflavin therapy [4,8, 11, 12]. *Leuconostoc mesenteroides*, *Lactococcus lactis*, *Limosilactobacillus fermentum*, *Lactiplantibacillus plantarum* and *Propionibacterium freudenreichii* have all been successfully treated with this technique [9,13,16].

In particular, a dairy product fermented with *Propionibacterium freudenreichii* demonstrated the ability to alleviate riboflavin deficiency in an animal model [17]. It is crucial to highlight that these strains of roseoflavin-resistant bacteria are spontaneous, non-genetically modified organisms. They could therefore be used to produce foodstuffs that are enhanced with vitamin B_2_ [10].

Innovative food products catered to particular demographics, such as the elderly, children, pregnant women, athletes, vegetarians and teenagers, could be developed with the concept of *in situ* riboflavin generation employing specific *B. subtilis* [11]. In this context, *B. subtilis* strains isolated from fermented African locust beans (a traditional condiment commonly used across West Africa) were exposed to roseoflavin, and the strains that overproduce riboflavin were isolated and utilized for the production of functional fermented African locust beans, with the objective that the technological qualities that these strains could provide in this condiment could help people who are riboflavin deficient.

The functional characteristics of fermented foods are primarily determined by their abundant content of micro-organisms possessing probiotic traits. Probiotics are described by the WHO and the Food and Agriculture Organization (FAO) of the United Nations as ‘live micro-organisms which, when administered in adequate amounts, confer a health benefit on the host’ [18]. Owing to their well-established health benefits, probiotics are being used more and more as ingredients in food items – which can be fermented foods or non-fermented products enhanced with probiotics – and as nutraceuticals in the manufacturing of food supplements [19].

This condiment has the potential to provide the host not only with health-promoting properties but also with increased *in situ* riboflavin production by delivering probiotic *B. subtilis* that overproduces vitamin B_2_ for *in situ* synthesis in the digestive tract.

In light of the aforementioned, the aim of this study was to carry out an *in vitro* screening on two riboflavin-overproducing strains, *B. subtilis* ACU-I11MR and ACU-I163MR strains, isolated from fermented African locust beans for their probiotic potential.

## Methods

### Chemicals, growth media and strains

Chemicals and growth media, unless specified, were from Sigma-Aldrich Pvt. Ltd. (St. Louis, MO, USA). Two different riboflavin-producing strains of *B. subtilis* were previously obtained from fermented African locust beans and identified through conventional methods. They were exposed to roseoflavin, and the overproducing strains (ACU-I11MR and ACU-I163MR) were obtained, which resulted in riboflavin concentrations of 0.0259*±*0.0077 mg l^−1^ and 0.0267*±*0.0013 mg l^−1^, respectively. *Escherichia coli*, *Salmonella enterica*, *Staphylococcus aureus* and *Aeromonas veronii* used for antibacterial study were obtained from the Microbiology Laboratory, Ajayi Crowther University, Nigeria. Growth media (nutrient broth and nutrient agar) were from Hi-Media Laboratories (India).

### Biosafety assessment

#### Haemolytic activity

Blood agar containing 5% (v/v) human blood free of antibiotics was used to assess the haemolytic activity. Each *B. subtilis* strain was cultured overnight, spread thinly over the blood agar surface and then incubated at 37 °C for 48 h. A halo formed around the inoculation line was used to categorize the results into γ-, α- and β-haemolysis. Particularly, green-coloured areas surrounding colonies denote α-haemolysis, whereas clear areas surrounding colonies signify β-haemolysis. Conversely, the lack of any zones surrounding the colonies denotes γ-haemolysis or no haemolytic activity.

#### Gelatinase activity

Ten microlitres of the overnight culture of *B. subtilis* isolates were put into nutrient agar that had been supplemented with 5% (w/v) gelatin. After that, the plates were incubated at 37 °C for 48 h. Following the incubation period, saturated ammonium sulphate solution was poured onto the plates. A positive result was shown by the formation of halos or clear zones surrounding the dots against the opaque background.

#### Biogenic amine production

By adding 0.5% (w/v) of the amino acid precursors, such as l-tyrosine disodium salt, l-tryptophan, l-lysine and l-histidine, to nutrient agar plates containing 0.06% bromocresol purple, the production of tyramine, tryptamine, cadaverine and histamine was determined. After being streaked on the plate, each *B. subtilis* colony was incubated for 3 days at 30 °C. The development of the purple hue signified the production of biogenic amines [20,22].

#### Antibiotic susceptibility test

The antibiotic susceptibility of the two *B. subtilis* strains was ascertained using the disc diffusion assay technique, which was implemented in accordance with the Clinical and Laboratory Standards Institute guidelines. Specifically, the entire surface of Mueller–Hinton agar plates was swabbed with overnight cultures of the *Bacillus* strains adjusted to 0.5 McFarland standard (to estimate the bacterium cell density). The antibiotics were aseptically applied to the surface of swabbed Mueller–Hinton agar plates. These included azithromycin (AZN, 15 µg), cefixime (ZEM, 5 µg), cefotaxime (CTX, 25 µg), ceftriaxone (CRO, 45 µg), cefuroxime (CXM, 30 µg), ciprofloxacin (CIP, 5 µg), erythromycin (ERY, 15 µg), gentamicin (GN, 10 µg), imipenem (IMP, 10 µg), levofloxacin (LBC, 5 µg), ofloxacin (OFL, 5 µg) and amoxicillin/clavulanate (AUG, 30 µg). After 24 h of incubation at 37 °C, zones of inhibition were observed on the plates and recorded [23].

### Probiotic properties

#### Acid tolerance test

Since pH tolerance affects the likelihood that an exogenous culture will survive in the gastrointestinal tract (GIT), it is an important factor to consider when choosing probiotic strains. Freshly made 10 ml nutrient broth adjusted to pH 2 and 3 using 0.1 M HCl was inoculated with a 2 ml aliquot of the *Bacillus* strains cultivated in nutrient broth overnight, and it was then incubated at 37 °C. Samples were taken at different times (0–3 h). Following collection, every sample was spread out onto nutrient agar plates and incubated at 37 °C for 24 h. For each tested pH condition, three biologically independent replicates were performed. The viable cells were counted, and their percentage of viability was expressed using the formula below:

$\text{Viability (\textbackslash{}\%)}=\left(\frac{N_{t}}{N_{0}}\right)\times 100;$

*Nt*=log FU at intervals 1, 2 and 3 h and

*No*=log at 0 h.

#### Bile salt tolerance

Similar to the acid tolerance test, 10 ml of freshly prepared nutrient broth, containing 0.3, 0.5 and 1% bile salt, was inoculated with a 2 ml aliquot of each *Bacillus* culture that had been grown overnight in nutrient broth and was incubated for 0–3 h at 37 °C. Every hour, samples were collected, spread onto nutrient agar plates with a glass rod and incubated at 37 °C for 24 h before counting the number of viable cells [23]. For each bile salt concentration, three biologically independent replicates were performed. The percentage survival of the organisms was calculated with the formula below:

$\text{Viability (\textbackslash{}\%)}=\left(\frac{N_{t}}{N_{0}}\right)\times 100;$

*Nt*=log at intervals 1, 2; and 3 h and

*No*=log c.f.u. at 0 h.

#### Phenol tolerance test

To assess the phenol tolerance of the *B. subtilis* strains, 100 µl of each 24-h-old *Bacillus* culture was inoculated into 900 µl of nutrient broth that contained 0.2 and 0.5% of phenol. After incubation for 24 h, the OD of the broths was measured at 600 nm [23]. The experiment was done in biologically independent triplicates. Values obtained were used to calculate the viability (%) using the formula below:

$\text{Viability (\textbackslash{}\%)}=\frac{{\text{OD}}_{24}}{{\text{OD}}_{0}}\times 100.$

#### Growth in different concentrations of sodium chloride and temperature

The ability of the *B. subtilis* strains to grow in variable temperatures (20, 37 and 42 °C) and sodium chloride (2 and 4%) was tested. The strains were cultured in nutrient broth overnight at 37 °C. They were then sub-cultured in fresh nutrient broth with a 1 : 100 inoculum, v/v, within the following parameters: temperature (20, 37 and 42 °C), salt (2 and 4% of NaCl, w/v) and ethanol (8 and 10%, v/v). The strains were cultivated for 8 h at 37 °C. The growth of each isolate was monitored at 4 and 8 h by measuring the OD_600 nm_ using a UV/visible (UV/VIS) spectrophotometer. At each temperature condition, three biological independent replicates were performed.

#### Auto-aggregation test

The auto-aggregation of the two *B. subtilis* strains was determined using the method described by Nwagu *et al*. [23]. A 24-h culture of the strains was centrifuged at 10 000 r.p.m. for 10 min to obtain the bacterial cell pellets. The cell pellets were washed twice with PBS and re-suspended with 3 ml of PBS. The resulting suspension was vortexed for 30 s, and 0.1 ml was withdrawn from the upper layer and mixed with 2.9 ml of PBS. The absorbance was taken after 0, 1, 2 and 3 h at 600 nm using a UV/VIS spectrophotometer. The experiment was done in biological independent triplicates. The auto-aggregation percentage was calculated with the formula below:

Auto-aggregation (%)=(1–A_t_/A_0_)×100;

A_t_=absorbance at 1, 2 and 3 h at 600 nm,

A_0_=absorbance at 0 h at 600 nm.

#### Cell hydrophobicity/bacterial adhesion to hydrocarbon

The cell hydrophobicity of the isolates was determined according to Lee *et al*. [24]. A 24-h culture of the *B. subtilis* strains was centrifuged at 10 000 r.p.m. for 3 min. The cell pellets were washed twice with PBS and re-suspended in 2 ml PBS. The absorbance of the final suspension was measured at 600 nm, and this served as the value for A_0_. The cell suspension was mixed separately with equal volumes of hydrocarbon ethyl acetate, acetone and xylene. Subsequently, the mixture was vortexed for 5 min and allowed to separate into two phases over a period of 30 min. Then, the absorbance of the aqueous phase was measured at 600 nm, which served as the value for A_1_. The experiment was done in biological independent triplicates. The cell hydrophobicity percentage was calculated using the formula below:

Hydrophobicity (%)=(1–A_1_/A_0_)×100.

#### Antibacterial activity

The standard agar well diffusion assay for broth culture of test pathogens and cell-free supernatant (CFS) was applied to assess the antibacterial activity of the two *Bacillus* strains. The test pathogens, Gram-negative bacteria (*A. veronii*, *E. coli* and *S. enterica*) and Gram-positive bacterium (*S. aureus*), were obtained from the stock culture of the Microbiology Laboratory, Ajayi Crowther University, Nigeria. An overnight culture of the test organisms adjusted to 0.5 McFarland standard (to estimate the bacteria cell density) was swabbed onto the entire Mueller–Hinton agar plates, after about 15–20 min, and wells of 5 mm diameter were bored/punched in the agar with a sterile cork borer. From the *Bacillus* isolates, the supernatant containing antimicrobials was extracted by centrifuging the cultures for 1 min at 13 000 r.p.m. Next, 100 µl of CFS was introduced into every bored well. The negative control was sterile water. In an upright position, the CFS was given about an hour to diffuse into the agar. The plates were incubated for 24 h at 37 °C in an inverted posture following full diffusion. The development of inhibition zones surrounding the wells served as a basis for determining the antibacterial activity of the bacterial isolates. Using a ruler, the diameter (measured in millimetres) of the clear inhibition zone surrounding the wells was recorded [25,26].

#### Biofilm formation

The *B. subtilis* strains were grown in nutrient broth at 37 °C for 24 h. The assessment of biofilm formation was conducted using NGM agar [Nutrient-Glycerol-Manganese sulphate agar; lycerol-manganese sulphate agar; nutrient agar supplemented with 1% (v/v) glycerol and 0.1 mM MnSO4] and NGM broth [nutrient broth supplemented with 1% (v/v) glycerol and 0.1 mM MnSO4]. For the agar method, each isolate was streaked on NGM agar plates and incubated at 37 °C for 72 h. For the broth method, an inoculum of 15 µl of each strain was inoculated to 15 ml of NGM broth and incubated at 37 °C for 24–48 h. The production of films at the NGM broth–air interface or the appearance of viscous and mucoid colonies on NGM agar indicated that the tested strains had formed biofilms [27].

#### Determination of proteolytic activity

Protease activity was determined on a solidiﬁed skim milk agar plate. An 8 mm-diameter well was made in the middle of the solidified plates using a sterile cork borer, an aliquot of 100 µl of 18 h culture was added to the well and plates were incubated at 37 ℃ for 72 h. The appearance of zones of clearance around the well indicated a positive result. Clear zones around each colony were measured in millimetres with a ruler.

#### Determination of amylase activity

The amylase test was conducted using starch agar (nutrient agar supplemented with 1% soluble starch). A line of streak of each bacteria isolate was made on the starch agar plates and incubated at 37 °C for 24 h. After the incubation period, the surface of the plates was flooded with iodine solution for 30 s. Excess iodine was drained off, and the presence of a clear zone around the line of bacterial growth was examined. A clear zone around the line of growth following the addition of iodine solution indicates the organism’s ability to produce amylase. Conversely, a blue, purple or black colouration of the entire medium indicates a negative result, signifying that the organism cannot produce amylase [28]. Clear zones around the colony were measured in millimetres with a ruler.

#### Exopolysaccharide production

Exopolysaccharide (EPS) production was assessed using the method described by Khalil *et al*. [29]. Each strain was streaked onto the surface of nutrient agar supplemented at 2% (w/v) with sucrose (Merck) plates and incubated at 37 °C for 48 h. The presence of colonies exhibiting mucoid or ropy features indicated a positive result [29,30].

### Molecular identification

The *B. subtilis* isolate with the best probiotic potential was further characterized molecularly. The genomic DNA was extracted using the Quick-DNA Fungal/Bacterial Miniprep Kit (Zymo Research, Catalogue No. D6005) according to the manufacturer’s instructions. After extraction, the quality and quantity of the extracted DNA were measured using a nanodrop (Thermo Scientific™ NanoDrop™ One Microvolume UV–Vis Spectrophotometer). The DNA was amplified by PCR with *gyrA*-F (5′ CAGTCAGGAAATGCGTACGTCCTT-3′) and *gyrA*-R (5′ GTATCCGTTGTGCGTCAGAGTAAC-3′) primers. The integrity of the DNA was assessed through electrophoresis on 1% agarose gels. Subsequently, the fragments were sequenced using the Nimagen Brilliant Dye™ Terminator Cycle Sequencing Kit V3.1, BRD3-100/1000, following the manufacturer’s instructions. DNA sequencing data were compared to the sequences of known strains using the Basic Local Alignment Search Tool database of the National Center for Biotechnology Information (NCBI). A similarity of greater than 98% was utilized as the criterion for species identification. The sequences were deposited in NCBI GenBank and accessioned.

### Statistical analysis

The findings in this study are shown as the mean±sd for each test, which was carried out in biological independent triplicate. A two-way ANOVA followed by Tukey’s post-hoc test was used to examine the differences between the two *B. subtilis* strains, with significance established at *P*<0.05. Microsoft Excel and GraphPad Prism version 10.2.0 were used to conduct statistical analyses.

## Results

### Biosafety assessment

*B. subtilis* ACU-I163MR and ACU-11MR were non-haemolytic and negative for gelatinase and biogenic amine activities.

The antibiotic susceptibility test showed that *B. subtilis* ACU-I163MR was susceptible to all the 12 antibiotics it was tested with, while *B. subtilis* ACU-I11MR was susceptible to 9 out of 12 antibiotics; it was resistant to CTX, ZEM and CRO (Table 1).

**Table 1.** Antibiotic susceptibility profile of the riboflavin-overproducing *B. subtilis* strains

Isolates/antibiotics	AUG	AZN	CTX	ZEM	CRO	CXM	CIP	ERY	GN	IMP	LBC	OFL
ACU-I163MR	S	S	S	S	S	S	S	S	S	S	S	S
ACU-I11MR	S	S	R	R	R	S	S	S	S	S	S	S

AUG, amoxicillin clavulanate (30 µg); AZN, azithromycin (15 µg); CTX, cefotaxime (25 µg); ZEM, cefixime (5 µg); CRO, ceftriaxone (45 µg); CXM, cefuroxime (30 µg); CIP, ciprofloxacin (5 µg); ERY, erythromycin (15 µg); GN, gentamicin (10 µg); IMP, imipenem (10 µg); LBC, levofloxacin (5 µg); OFL, ofloxacin (5 µg).

### Probiotic properties

#### Acid tolerance

**Fig. 1.**
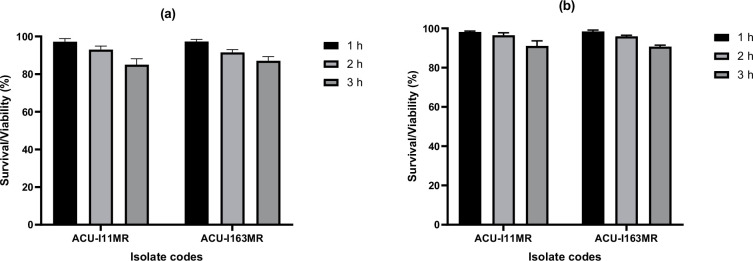
Survival of the riboflavin-overproducing *B. subtilis* strains under exposure to (**a**) pH 2 and (**b**) pH 3.

After 3 h of incubation, *B. subtilis* ACU-I163MR and ACU-I11MR survived at pH 2 and 3. (Fig. 1). During the 3 h of incubation, the survival rate was higher at pH 3 than it was at pH 2. The ultimate survival rates at pH 2 were 85.15 and 87.09% with *B. subtilis* ACU-I163MR discovered to be the most acid-tolerant strain (Fig. 1a). At pH 3, the rates were marginally higher, coming in at 91.11 and 90.72% (Fig. 1b). There were no discernible variations in the survival rate between the two strains (*P*>0.05).

#### Bile tolerance

**Fig. 2.**
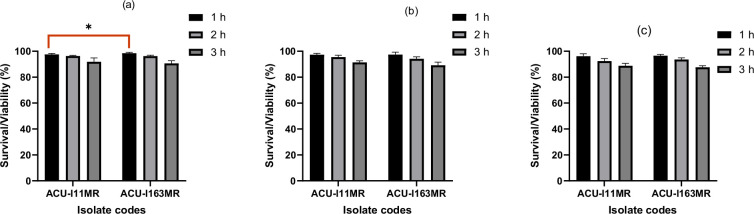
Viability of the riboflavin-overproducing *B. subtilis* strains after exposure to different concentrations of bile salt: (**a**) 0.3%, (**b**) 0.5% and (**c**) 1%. *P<0.05 as determined by two-way ANOVA with Tukey’s multiple comparisons test (*n*=3).

Both isolates can withstand different bile salt concentrations of 0.3, 0.5 and 1% while retaining an 87% survival rate (Fig. 2a–c). The highest survival rate was noted at a bile salt concentration of 0.3%, while the lowest was noted at a concentration of 1%. The survival rate falls as bile salt content rises. Both strains (ACU-I11MR and ACU-I163MR) survived well at a high concentration of bile salt (1%) after 3 h exposure, yielding 88.82 and 87.64%, respectively. There was no significant difference (*P>*0.05) in survival rates observed between both strains after 3 h exposure to 0.5 and 1.0%. However, statistically, *B. subtilis* ACU-I163MR had a higher survival rate (*P<*0.05) of 98.44% after 1 h exposure to 0.3% bile salt than ACU-I11MR, which had 97.56%.

#### Phenol tolerance

**Fig. 3.**
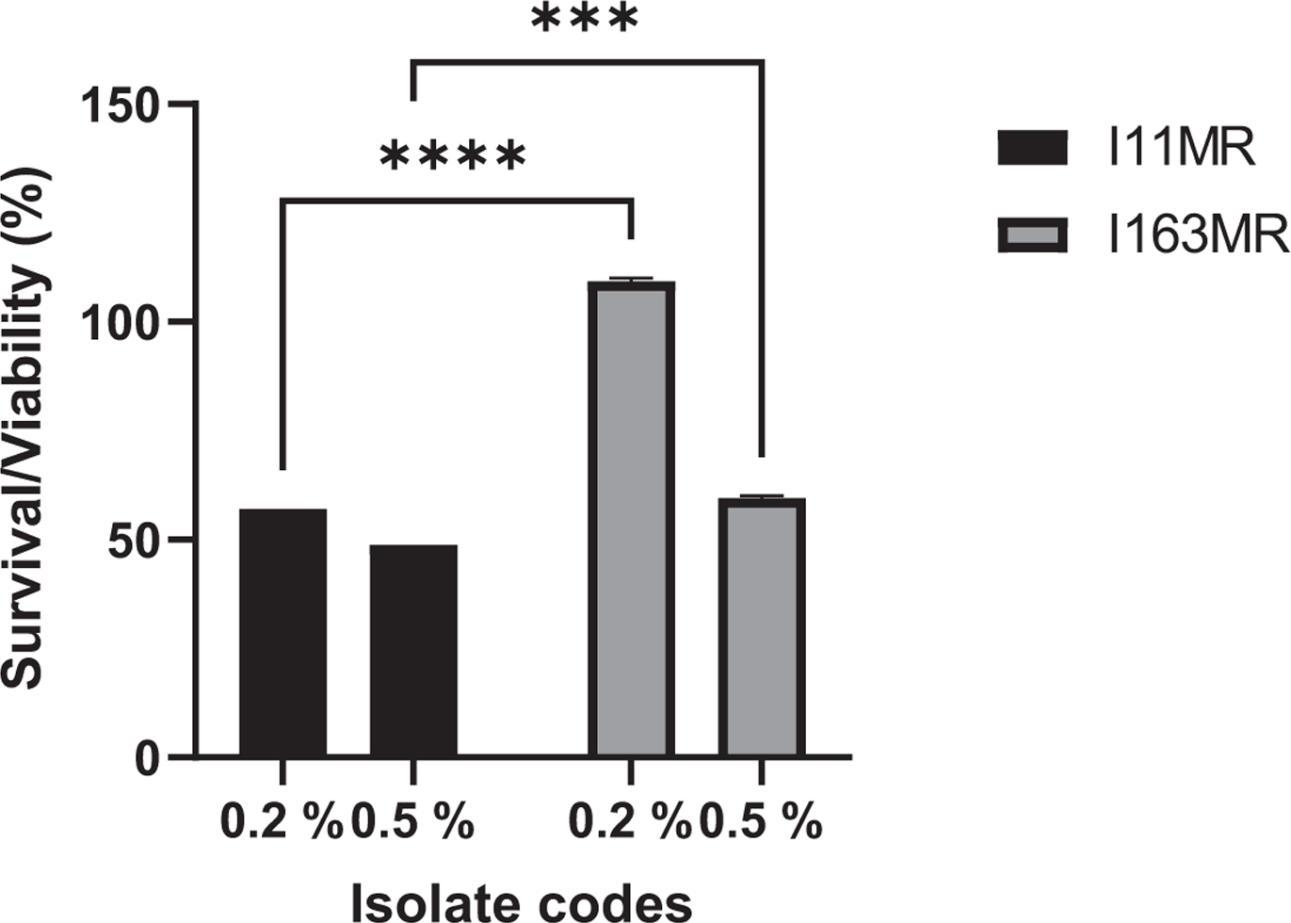
Viability of the riboflavin-overproducing *B. subtilis* strains after exposure to 0.2 and 0.5% phenol. ^***^P< 0.001, ^****^P< 0.0001 as determined by two-way ANOVA with Tukey’s multiple comparisons test (*n*=3).

After 24 h, *B. subtilis* ACU-I163MR showed the highest viability (109.28%) in nutrient broth containing 0.2% phenol (Fig. 3). The viability of *B. subtilis* ACU-I11MR was significantly lower (*P*<0.0001) exhibiting 57.00%. Likewise, in the 0.5% phenol broth, both strains exhibited good viability (Fig. 3). Viability percentages 48.80 and 59.52% were recorded for *B. subtilis* ACU-I11MR and ACU-I163MR, respectively (*P*<0.0001). The strains were tolerant to both phenol concentrations with relatively lower stability observed in higher phenol concentrations (0.5%).

#### Growth in varying temperatures and NaCl concentrations

**Fig. 4.**
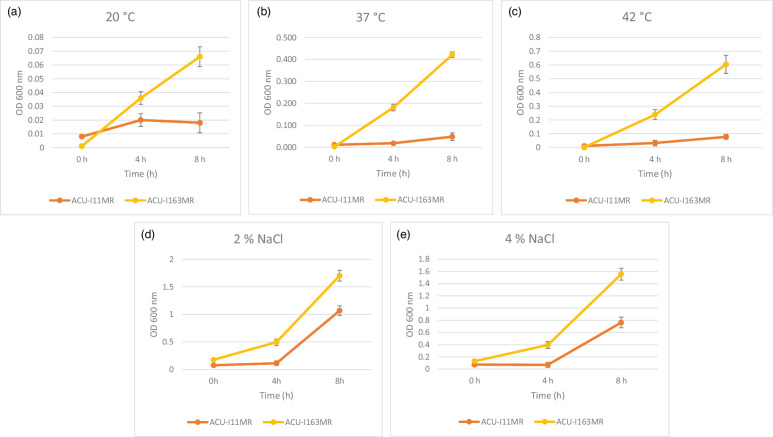
Growth of riboflavin-overproducing *B. subtilis* strains at different temperatures and NaCl concentrations: (**a**) 20 °C, (**b**) 37 °C, (**c**) 42 °C, (**d**) 2% NaCl and (**e**) 4% NaCl.

The strains were capable of growing at 20, 37 and 42 °C. Furthermore, they showed differing degrees of tolerance to different sodium chloride concentrations; the growth rate of strains dropped as the NaCl levels rose from 2 to 4%. Compared to the other strain, *B. subtilis* ACU-I163MR was generally more thermostable and halotolerant (Fig. 4a–e).

#### Auto-aggregation

**Fig. 5.**
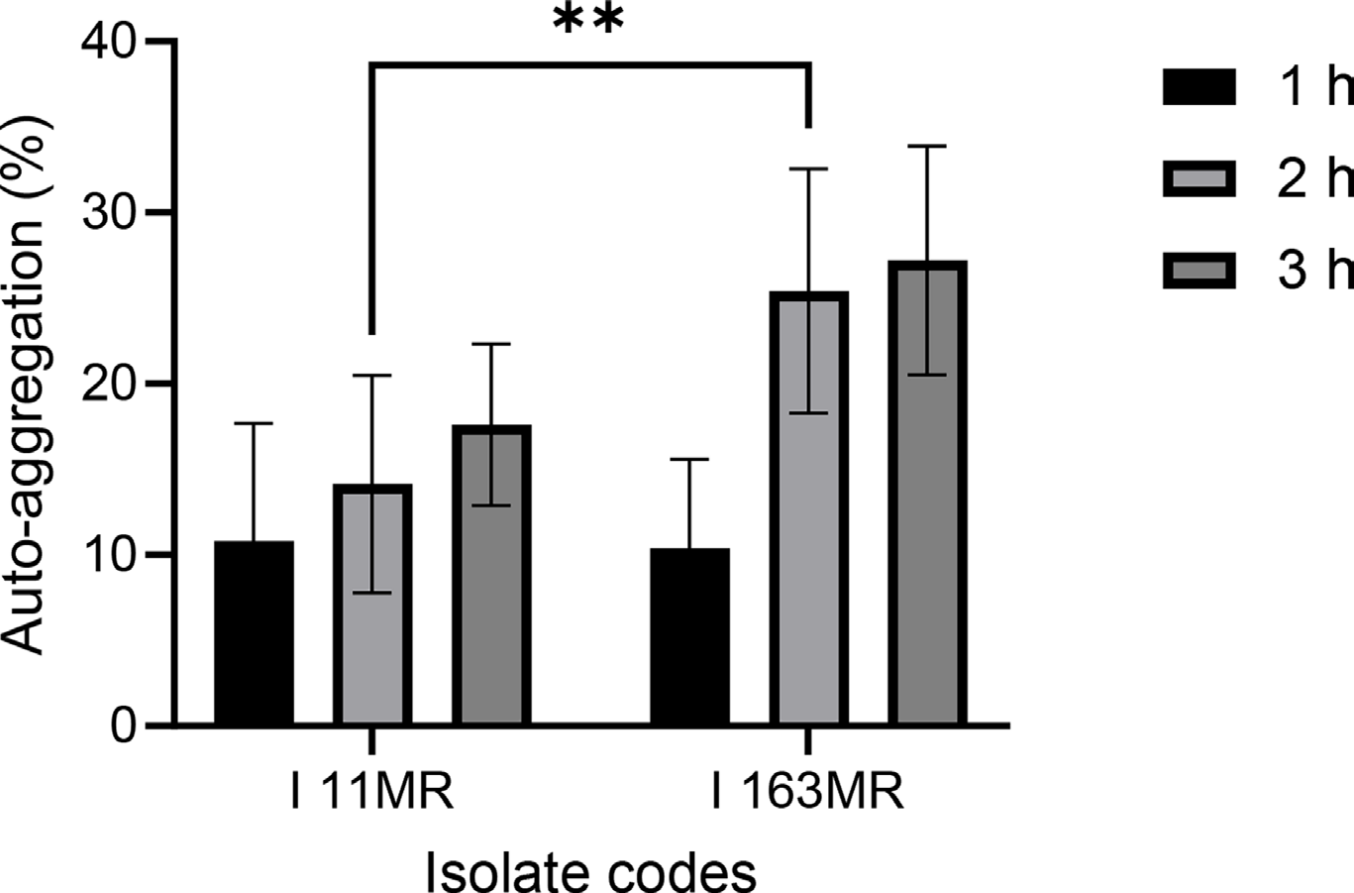
Percentage (%) of auto-aggregation obtained for the riboflavin-overproducing *B. subtilis* strains. ^**^P< 0.01 as determined by two-way ANOVA with Tukey’s multiple comparisons test (*n*=3).

The two *B. subtilis* strains tested were observed to have a considerable cell adhesion ability after 3 h. Specifically, *B. subtilis* ACU-I163MR was observed to have an auto-aggregation percentage of 10.39% after 1 h; this increased to 25.41% after 2 h and reached 27.20% after 3 h (Fig. 5). A similar trend was observed with other *B. subtilis* strains, i.e. increase in auto-aggregation percentage with increasing time. However, after 2 h, the auto-aggregation percentage of *B. subtilis* ACU-I11MR was significantly lower (*P*<0.01) exhibiting 14.14% compared to ACU-I163MR.

#### Cell hydrophobicity

**Fig. 6.**
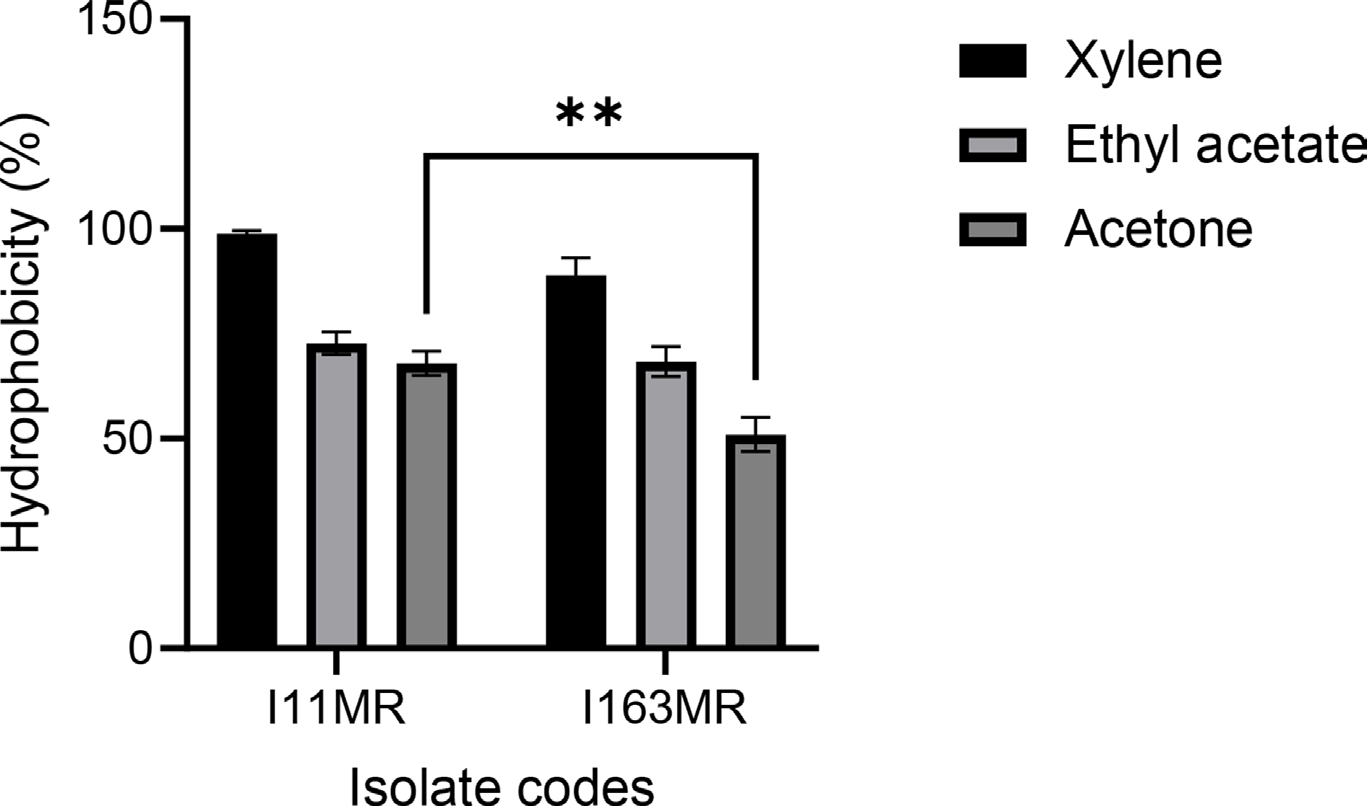
Percentage (%) of cell hydrophobicity obtained for the riboflavin-overproducing *B. subtilis* strains. ^**^P< 0.01 as determined by two-way ANOVA with Tukey’s multiple comparisons test (*n*=3).

Microbial adhesion to non-polar solvents (xylene, ethyl acetate and acetone) reflects cell surface hydrophobicity. In this study, the two *B. subtilis* strains (ACU-I11MR and ACU-I163MR) showed higher adhesion to xylene, exhibiting 98.84 and 88.95%, respectively, compared to other non-polar solvents. For the other non-polar solvents, the percentage of hydrophobicity ranged from 50.98 to 72.74% (Fig. 6).

#### Antibacterial activity

The antibacterial activity of the neutralized cell-free crude supernatant against indicator strains of *S. aureus*, *A. veronii*, *S. enterica* and *E. coli* showed that the antibacterial spectrum of the test *Bacillus* strains was similar (Table 2) with their inhibition zones ranging from 0 to 20 mm. *B. subtilis* ACU-I11MR and ACU-I163MR showed antibacterial activity against all four bacterial pathogens and exhibited the largest inhibition diameter (15 and 20 mm) against *E. coli*.

**Table 2.** Antibacterial activity of riboflavin-overproducing *B. subtilis* strains

Pathogens	Diameter of zone of inhibition (mm)	
	ACU-I11MR	ACU-I163MR
*Escherichia coli*	15	20
*Staphylococcus aureus*	12	10
*Salmonella enterica*	8	8
*Aeromonas veronii*	15	10

#### Biofilm formation

Both tested *B. subtilis* strains exhibited the capacity to produce diverse forms of biofilms with varying thickness and density. These biofilms were observed on the surface of NGM agar as well as on the inner walls of the test tubes containing NGM broth.

#### Determination of proteolytic and amylase activity

The proteolytic activity results of the *B. subtilis* strains are indicated by zones of clearance on the skim agar plates. Isolates ACU-I11MR and ACU-I163MR displayed proteolytic zones of clearance of 8 and 12 mm, respectively. The zone of hydrolysis was used to determine amylase activity on starch agar, and the *B. subtilis* strains exhibited 10 and 12 mm hydrolysis zones, respectively.

#### EPS production

When a colony was picked up using a bacteriological loop, the two *B. subtilis* strains had the phenotype ‘ropy’ characteristic, which was visible as a thin filament.

### Molecular identification of *Bacillus*

*B. subtilis* ACU-I163MR, being the strain with the best probiotic potential, was identified at the molecular level and designated as *B. subtilis*. The nucleotide sequences have been deposited in the NCBI database under the GenBank accession number PP886124.

## Discussion

The present study aimed to assess the probiotic potential of two riboflavin-overproducing *B. subtilis* strains isolated from traditional fermented African locust beans. While other micro-organisms such as *Ashbya gossypii* and *Candida famata* are industrially relevant in riboflavin production, *B. subtilis* offers advantages characterized by rapid growth, minimal nutritional requirements and facile product extraction from fermentation broth [8].

A series of tests is necessary to choose micro-organisms with probiotic qualities. The strains analysed must adhere to the FAO/WHO’s *in vitro* evaluation guidelines [31]. The safety examination, which ensures no risk to human health, is one of the probiotic tests. Haemolysin or harmful byproducts that could lyse red blood cells were absent from the two strains of *B. subtilis* under study, ACU-I11MR and ACU-I163MR; they do not produce gelatinase and do not show biogenic amine formation capabilities. The safety of food products can be jeopardized by some bioactive substances, including biogenic amines, which are often present in fermented foods due to bacterial decarboxylation of specific amino acids by fermentative bacteria [32]. The consumption of foods containing high concentrations of these biogenic amines can result in food intoxication and other adverse health effects [32,33]. Gelatinase, biogenic amines and haemolytic activities in micro-organisms are usually considered virulence factors [34]. Hence, they are of public health concern, and their absence is normally recommended for strains intended as probiotics.

Antibiotic sensitivity is another important factor to take into account when assessing a probiotic. Although *B. subtilis* strains have been designated as Qualified Presumption of Safety or Generally Recognized as Safe, there is a chance that they could act as reservoirs for resistance genes [35,36]. The two *B. subtilis* strains examined in this study were responsive to the antibiotics that were tested (Table 1), proving that they do not exhibit atypical resistance to clinically significant antimicrobials. This supports the EFSA (The European Food Safety Authority) report [37]. Likewise, other studies have reported a similar sensitivity profile for *Bacillus* probiotic strains [23,27, 38, 39].

When choosing a probiotic, one of the most crucial functional characteristics to take into account is its capacity to withstand the severe circumstances of the GIT, including the acidity of the stomach environment, and its persistence in the intestinal habitat [31]. The probiotic strain must be able to withstand high levels of acidity and bile salt in the GIT, with little to no loss of viability in order to establish successive stable viability [40]. In this study, pH levels of 2.0 and 3.0, corresponding to that of the stomach, and bile salts in the small intestine were evaluated [0.3, 0.5 and 1.0% (w/v)]. Both strains tolerated these conditions. The two strains, *B. subtilis* ACU-I11MR and ACU-I163MR, were shown to have strong resistance to pH 2 and 3, as evidenced by survival rates that exceeded 85 and 87%, respectively. Likewise, the bacteria grew and survived in the bile salt at concentrations of 0.3, 0.5 and 1.0%, with a survival rate exceeding 87%. Numerous researchers have noted that strains of this species are tolerant of various pH levels [23,24, 27, 38].

Phenols are recognized as toxic metabolites released when certain aromatic amino acids from food and internal proteins are deaminated by bacteria. These substances have bacteriostatic properties [38]. After being incubated for 24 h in a medium containing 0.2% phenol, *B. subtilis* ACU-I163MR showed the highest viability (103.28%) and 59.92% in a medium containing 0.5% phenol. Likewise, Dabiré *et al.* [27] observed that *Bacillus* strains *Bacillus dakarensis*, *Bacillus cereus*, *Bacillus benzoevorans*, *B. subtilis*, *Bacillus cabrialesii* and *Bacillus tequilensis* were typically found to be moderately tolerant to 0.4% phenol. The likelihood of survival for bacteria is higher in those that have developed a tolerance to phenols than in those that do not.

Evaluating the effectiveness of a probiotic largely depends on its capability to endure fluctuating internal body temperatures because the bacteria may experience shock upon consumption [41]. Furthermore, saline stress during microbial development can cause turgor pressure and water outflow to decrease, which can negatively impact the physiology of the cell as a whole and the production of necessary components [23]. The two strains evaluated in this study can survive at 20, 37 and 45 °C. Furthermore, they showed differing degrees of tolerance to sodium chloride concentrations of 2 and 4%. The literature has documented *Bacillus* species’ resistance to both heat and salinity stressors [23,27]. The capacity of these strains to produce spores may be the reason for their remarkable resistance to gastrointestinal stressors. This capacity permits the creation of a four-layered protective membrane that helps insulate the bacteria from an unfavourable GIT environment [38].

Probiotics need to be able to adhere to the host’s digestive system in order to do their job. Auto-aggregation and cell hydrophobicity are two indirect measures of adhesion. Probiotics should have auto-aggregation because it guarantees that they reach a high density in the intestine, shielding them from stressors [42], facilitating their interaction with the host, enhancing their ability to adhere to surfaces and colonize the GIT and producing advantageous outcomes like squelching possible pathogens [43]. Auto-aggregation percentages equal to or lower than 10% correspond to strains that are unable to auto-aggregate, while strains with percentages higher than 10% can naturally auto-aggregate [44]. In this study, the two strains presented auto-aggregation values higher than 10% similar to the reports by several authors for the same species [38,40, 45]. Cell aggregation facilitates improved adhesion to digestive system cells and a denser environment in the gut [38]. The relationship between the bacterial surface and the intestinal epithelial cells has been linked to hydrophobicity in probiotics. It is dependent upon non-polar compounds found in the cell wall and membranes, such as polysaccharides and glycoproteins [30]. This attribute serves as a crude gauge of how well probiotics adhere and colonize [46]. Therefore, while its assessment enables the surface of the strains to be characterized in terms of their ability to interact with intestinal cells and mucosa, it is not a requirement for robust adherence [47]. Both *B. subtilis* ACU-I11MR and ACU-I163MR showed a higher affinity for xylene (98.84 and 88.95%) and a lesser affinity for ethyl acetate and acetone ranging from 50.98 to 72.74%, similar to the reports of other authors [23,24, 27]. Because of their degree of hydrophobicity and capacity for auto-aggregation, these strains have demonstrated encouraging probiotic outcomes. The multi-layered protein sheath encasing the *Bacillus* species spores may be responsible for the hydrophobic characteristics of the isolated strains [38].

Antibacterial activity is a vital factor in the selection of probiotic bacteria since these bacteria fight foodborne pathogens. According to the findings from this study, the cell-free supernatant (CFS) from *B. subtilis* ACU-I11MR and ACU-I163MR strains showed an inhibitory effect against the four bacterial pathogens tested. These findings align with other authors’ reports on these species [38,45, 48]. These organisms could potentially be used in food preservation since their presence in food products may decrease the ability of pathogens to survive. *B. subtilis* is known to produce a variety of metabolites, including bacteriocin and organic acids, which have been linked to its inhibitory behaviour [49,50].

The findings of this study demonstrated that both *B. subtilis* strains were able to produce amylase and protease. A probiotic strain has to produce hydrolytic enzymes like amylase and protease in order to function as an efficient food fermenter. These enzymes are essential for dissolving complex dietary polymers into simpler substances like oligosaccharides, peptides and amino acids. After going through additional biological interactions, these simpler molecules can generate organic acids and other chemicals that affect flavour and have health advantages [27].

The two riboflavin-overproducing *B. subtilis* examined in this study are capable of producing biofilm. Despite being implicated in most chronic illnesses, biofilms play significant roles in biocontrol mechanisms [51]. Vegetative cells that attach as a biofilm and colonize the surface of the intestinal mucosa proliferate when food containing *Bacillus* spores is consumed, and these spores germinate in the intestinal tract [24]. Therefore, these biofilms aid in the attachment of *Bacillus* spp. to intestinal epithelial cells, enhancing their persistence and multiplication on the intestinal mucosa [52] where they inhibit entero-pathogen adhesion and provide probiotic benefits to the host [24].

One of the most popular techniques for identifying the EPS production phenotype is the visual screening of colonies on a solid culture medium. The carbon and nitrogen sources in the environment affect the formation of EPS, and sucrose is a sugar that promotes the growth of EPS-producing colonies [53]. Both of the *B. subtilis* strains used in this investigation had a ropy phenotype, suggesting that they are capable of producing EPSs. These species, when cultivated on media containing sucrose, have been reported by other authors to form colonies with a ropy shape [45,54, 55]. EPS is produced by probiotics on their cell membrane, and they might appear as paracrystalline layers or capsules. These EPS function as barriers that shield cells from a variety of stressors, including phagocytosis, phage assaults, desiccation and osmotic stress. Furthermore, because of their EPS, probiotics have therapeutic uses in areas such as immunomodulation, antibacterial, anti-inflammatory, antioxidant, anticancer and cholesterol lowering [56].

Given the above properties, the riboflavin-overproducing strains in this study can be used in the fermentation of various foods to obtain riboflavin naturally enhanced products that also provide probiotic benefits. This can improve gut health and also address hidden hunger (micronutrient deficiencies) in regions where fortified foods are scarce or unaffordable. On the industrial level, they can be employed to develop natural vitamin supplements containing bioavailable riboflavin, offering a safer, more natural alternative to the synthetic prototype.

In this study, while the riboflavin-overproducing *B. subtilis* strains from ‘iru’ demonstrate promising potential for food fortification and industrial applications, the probiotic characteristics of these strains require further investigation. In particular, *in vivo* testing is essential to confirm their ability to survive and colonize the GIT, interact beneficially with the native gut microbiota and promote host health. Future studies should focus on conducting animal or human trials to evaluate the strains’ effects on nutrient absorption, immune modulation and overall gut health. These insights will be crucial for validating the strains’ probiotic efficacy and determining their suitability for commercial probiotic products or functional foods.

## Conclusions

Nowadays, there is a growing interest in functional foods with possible health advantages, where micro-organisms such as probiotics play important roles. In this context, the current study reveals that the two riboflavin-overproducing *B. subtilis* isolated from fermented African locust beans (ACU-I11MR and ACU-I163MR) were all ascertained to be safe and showed a strong ability to tolerate extreme environmental conditions, including low pH (2 and 3), bile salts (0.3, 0.5 and 1.0%) and phenol (2 and 4%) with different survival rates. The strains also exhibited cell surface hydrophobicity and auto-aggregation adhesion properties with their CFS having antibacterial activity against tested pathogenic strains. Additionally, they are capable of forming biofilms, which suggests that they may be able to colonize the intestines. They are good producers of the enzyme amylase and protease and EPSs. However, *B. subtilis* ACU-I163MR exhibited the best probiotic potential. Therefore, *B. subtilis* ACU-I163MR is a promising probiotic candidate with riboflavin production features, which could be exploited to produce riboflavin-rich *Bacillus*-fermented functional foods.

## Supplementary material

10.1099/acmi.0.000883.v3 Uncited Supplementary Material 1.

## Abbreviations

AUG: amoxicillin/clavulanate
AZN: azithromycin
CFS: cell-free supernatant
CIP: ciprofloxacin
CRO: ceftriaxone
CTX: cefotaxime
CXM: cefuroxime
EFSA: The European Food Safety Authority
EPS: exopolysaccharide
ERY: erythromycin
FAO: Food and Agriculture Organization
GIT: gastrointestinal tract
GN: gentamicin
IMP: imipenem
LBC: levofloxacin
NCBI: National Center for Biotechnology Information
OFL: ofloxacin
UV/VIS: UV/visible
WHO: World Health Organization
ZEM: cefixime
