# Supplementary material for: Probiotic potential of riboflavin-overproducing Bacillus subtilis ACU-I163MR and ACU-I11MR, isolated from fermented African locust beans
Source: Access Microbiol. 2025 Jan 28;7(1):000883.v3. doi: 10.1099/acmi.0.000883.v3 (PMC12282050; doi:10.1099/acmi.0.000883.v3)
Supplement: Uncited Supplementary Material 1. [file acmi-7-00883-s001.pdf]

Supplementary Table S1. Zone of diameter (mm) for antibiotic susceptibility profile of the riboflavin-overproducing *Bacillus subtilis* strains

Isolates/ Antibiotics	AUG	AZN	CTX	ZEM	CRO	CXM	CIP	ERY	GN	IMP	LBC	OFL
ACU-I163MR	28	25	22	22	26	23	28	27	28	20	24	32
ACU-I11MR	22	24	14	14	16	22	22	15	20	18	20	30

Supplementary Table S2. Survival (%) of the riboflavin-overproducing *B. subtilis* strains under exposure to pH 2

Isolate codes	1 h	2 h	3 h
ACU-I11MR	97.21877 ± 1.369	93.00135 ± 1.570	85.04718 ± 2.571
ACU-I163MR	97.29038 ± 0.919	91.55965 ± 1.253	87.09117 1.807

Supplementary Table S3. Survival (%) of the riboflavin-overproducing *B. subtilis* strains under exposure to pH 3

Isolate codes	1 h	2 h	3 h
ACU-I11MR	98.20187 ± 0.397	96.47308 ± 1.080	91.11284 ± 2.114
ACU-I163MR	98.43222 ± 0.570	95.95765 ± 0.419	90.7229 ± 0.629

Supplementary Table 4. Viability (%) of the riboflavin-overproducing *Bacillus subtilis* strains after exposure to 0.3% bile salt

Isolate codes	1 h	2 h	3 h
ACU-I11MR	97.55925 ± 0.574	96.31071 ± 0.432	91.85995 ± 2.437
ACU-I163MR	98.43756 ± 0.583	96.20328 ± 0.628	90.65556 ± 1.675

Supplementary Table 5. Viability (%) of the riboflavin-overproducing *Bacillus subtilis* strains after exposure to 0.5% bile salt

Isolate codes	1 h	2 h	3 h
ACU-I11MR	97.36058 ± 0.853	95.47531 ± 1.202	91.36646 ± 1.053
ACU-I163MR	97.43846 ± 1.500	94.23876 ± 1.223	89.24274 ± 1.940

Supplementary Table 6. Viability (%) of the riboflavin-overproducing *Bacillus subtilis* strains after exposure to 1.0% bile salt

Isolate codes	1 h	2 h	3 h
ACU-I11MR	96.20636 ± 1.597	92.36357 ± 1.672	88.81471 ± 1.535
ACU-I163MR	96.54894 ± 0.811	93.58702 ± 1.149	87.64287 ± 1.019

Supplementary Table 7. Viability (%) of the riboflavin-overproducing *Bacillus subtilis* strains after exposure to 0.2 % and 0.5 % phenol

Isolate codes	ACU-I11MR	ACU-I163MR
0.20%	57 ± 0.0	109.2834 ± 0.609
0.50%	48.80383 ± 0.0	59.51662 ± 0.427

Supplementary Table 8. Percentage (%) of auto-aggregation obtained for the riboflavin-overproducing *B. subtilis* strains

Isolate codes	1 h	2 h	3 h
ACU-I11MR	10.82461 ± 5.594	14.13488 ± 5.191	17.61083 ± 3.853
ACU-I163MR	10.3941 ± 4.246	25.41033 ± 5.819	27.20038 ± 5.458

Supplementary Table 9. Percentage (%) of cell hydrophobicity obtained for the riboflavin-overproducing *B. subtilis* strains

Isolate codes	Xylene	Ethyl acetate	Acetone
ACU-I11MR	98.83466 ± 0.562	72.73647 ± 2.199	67.9394 ± 2.335
ACU-I163MR	88.94865 ± 3.310	68.34792 ± 2.909	50.97635 ± 3.307

Supplementary Table 10. Growth of riboflavin-overproducing *B. subtilis* strains at 20 °C

Isolate codes	0 h	4 h	8 h
ACU-I11MR	0.008 ± 0.0002	0.02 ± 0.004	0.018 ± 0.0072
ACU-I163MR	0.001 ± 0	0.036 ± 0.0046	0.066 ± 0.007

Supplementary Table 11. Growth of riboflavin-overproducing *B. subtilis* strains at 37 °C

Isolate codes	**0 h**	**4 h**	**8 h**
ACU-I11MR	0.011 ± 0	0.018 ± 0.0015	0.048 ± 0.0172
ACU-I163MR	0.002 ± 0	0.181 ± 0.0015	0.422 ± 0.0132

Supplementary Table 12. Growth of riboflavin-overproducing *B. subtilis* strains at 42 °C

Isolate codes	0 h	4 h	8 h
ACU-I11MR	0.011 ± 0.008	0.032 ± 0.02	0.077 ± 0.018
ACU-I163MR	0.001 ± 0.001	0.239 ± 0.036	0.604 ± 0.066

Supplementary Table 13. Growth of riboflavin-overproducing *B. subtilis* strains in 2 % NaCl.

Isolate codes	0h	4h	8h

ACU-I11MR	0.076 ± 0.0012	0.113 ± 0.0143	1.068 ± 0.0872
ACU-I163MR	0.176 ± 0.0242	0.495 ± 0.0565	1.702 ± 0.0972

Supplementary Table 14. Growth of riboflavin-overproducing *B. subtilis* strains in 4 % NaCl.

Isolate codes	0 h	4 h	8 h
ACU-I11MR	0.072 ± 0.002	0.069 ± 0.0152	0.762 ± 0.078
ACU-I163MR	0.128 ± 0.022	0.393 ± 0.0544	1.554 ± 0.0968
